# My kuru adventure

**DOI:** 10.1098/rstb.2008.4031

**Published:** 2008-11-27

**Authors:** Werner H. Stöcklin

**Affiliations:** Albert Oeri Strasse 14CH-4125 Riehen, Basel, Switzerland

## 1. 1962

During my first employment as a Medical Officer in the Public Health Service of the Territory of Papua and New Guinea, I was, after an induction period in Goroka, offered a posting to Okapa for several months. I was accompanied by my wife Theresa and our 18-month-old son. The pilot who flew us there in a Cessna seemed to feel pity for the young family he had to carry to ‘such a remote place between clouds and forests—among tribes of cannibals, all suffering from the laughing death’. He did not realize what a privilege it was for us to be sent to Okapa at this early stage of kuru research. And we of course did not yet realize it either.

To make a long story short: we loved Okapa, its surroundings and most of the dark and white people we met. There was never a dull moment to make us miss civilization. And as for cannibalism: the new law had encouraged the Fore people for more than 2 years to give up eating their deceased relatives. It was a good feeling for me to be now the ‘Officer-in-Charge’ (OiC) of ‘PHD Okapa’ (the Public Health Department in Okapa), though the hospital consisted mainly of old grass-thatched buildings, being gradually replaced by modern constructions.

During the first two weeks, we did not encounter any kuru patients, as they were normally not brought to the hospital. But a kerosene-fed deep freezer had been placed in our house and it had a special space ‘for kuru brains only’, reminding us daily that kuru was a sad and deadly reality. Besides, nearly two dozen little kuru orphans, carefully looked after by Sister Maria Horn and by older siblings (the so-called ‘watchmen’), lived in a new building below the hospital after having lost their poor mothers from the ‘laughing death’ a short while after their birth.

Soon we met some of the kuru researchers at Okapa. Our favourite neighbours on a nearby hill were ‘the Alpers’—Michael Alpers and his family—unfortunately, most of the time away out in the bush. And we chatted with Bob and Shirley Glasse (Shirley later became Lindenbaum), the anthropologists involved in the social aspects of kuru. Last but not least, on rare occasions, we had the pleasure of the company of D. Carleton Gajdusek, the never tired, friendly kuru-guru. ‘Masta Galten’ (or ‘Kaoten’) was highly respected throughout the kuru region and was admired not only as a communicative all-round genius but also as a ‘cultural hero’.

Joining Mike Alpers on a routine patrol, I finally had a chance to meet a few female kuru victims in their villages. They all were in stage 2 or 3, and it was most impressive to see how cooperative and even joyful they were during their neurological check-up and how well they seemed to accept their disorders.

Then I heard about kuru sorcery, a subject that I find continually stimulating, something between medicine and ethnology, exactly what I had been longing for when I followed Prof. Alfred Bühler's lectures on cultural anthropology (as a special ‘escape’ during my medical studies in Basel). Soon I discovered that aid post orderly Tarubi (whom I knew as ‘Trilby’), the ‘dokta’ at the Purosa Aid Post (South Fore), was an expert in the subject, not as a sorcerer but as an informant, who had the traditional knowledge of his ‘tribe’ at his fingertips. I spent many hours with this intelligent young man and wrote down what he explained to me in his simple untwisted Pidgin.

As a special highlight, he organized for me an unforgettable medical patrol to the Moraei Kukukuku (Anga), living ‘next door’ on the slopes and ridges beyond the Lamari River. Their first contact with ‘the government’, represented by Patrol Officer Jack Baker, D. C. Gajdusek and some police, dated only a few years back and they still lived as uncontrolled, free ‘Stone Age’ people in a romantic world of yesterday. Carleton used to visit them often and knew them well.

Tarubi's explanations about kuru sorcery among the South Fore people were fascinating. I was astonished to hear how much ‘logic’ there was hidden behind all the strange thoughts that were embracing this subject. However, this paper is probably not the right place to go into endless technical details of sorcery, divination and curative efforts. Nevertheless, it may be interesting to know that any grown-up Fore male could have been able to perform deadly kuru sorcery, provided he had a sorcerer's stone and knew the complicated procedures with the most harmful spells, presupposing also that he was smart enough to collect secretly some dirty stuff from his future victim—and, finally, on condition that he was willing to follow a private course in kuru sorcery given by an expert. These detailed instructions had to be concluded with a final exam. If the teacher was satisfied with the results, he proudly called his candidate a *kanarayagara* (a good man). If the exam was not successful and the designated kuru victim did not get sick at all, the title for the useless newcomer was just *kasaua* (a potato man).

## 2. 1979

The wonderful experience of our posting to Okapa in 1962 was only of four-month duration. The rest of our term was spent in the completely different areas of the mighty Sepik River. We returned to Papua New Guinea again for colourful employment as OiC of PHD Maprik, 1969–1970. But it was not until 1979 that I was able to return to the kuru region for a few days, accompanied by my eldest son, Merrill, who had felt so much at home at Okapa some 17 years earlier. With Wendy Alpers' friendly help we were allowed to join the research team visiting some—in the meanwhile becoming increasingly rare—kuru victims in their villages. The patients we met were women of an advanced age group, clean and nicely dressed for their routine stage 2 and 3 check-ups. Their behaviour during the neurological examination was, though cooperative, slightly different from what I had seen years ago. I missed the sorrowless amusement that formerly appeared to be typical for the ‘laughing death’.

The mysteries of kuru had meanwhile been analysed at the highest scientific levels. And D. C. Gajdusek had been honoured with the Nobel Prize in 1976. Kuru was undoubtedly an infectious disease (with often extremely long incubation periods) spread out among the Fore and some neighbouring groups by their traditional endocannibalism, the habit of eating deceased relatives, even when they had died of kuru. All the medical and social questions regarding kuru found their appropriate answer with these explanations.

Even some of the Fore seemed to have been convinced now that germs might be involved in the kuru disease. Nevertheless, this belief was only partial. After an interesting beer and candlelight discussion with a local research assistant and two other Fore experts, I was sure that sorcery still had its place in their partially modernized world view. For them, sorcery could unveil all the kuru queries and mysteries just as well, and besides it offered patients a mild hope to get cured. For them, it was still evident that kuru had spread from one village to the other just following the secret tracks of sorcery knowledge. Kuru never reached the Kukukuku people because they were entirely ignorant and uninterested as far as this sorcery was concerned. The fact that kuru mainly used to kill women and children was not surprising, as it was much more difficult to perform sorcery against adult males. On the background of sorcery, it was also easy to understand that the number of new kuru victims had been gradually reduced in recent years. Sorcery had been forbidden by the government and, in addition, the sorcerers were getting discouraged by the great number of people condemning their intolerable activities. And why, after all, could kuru not be treated or cured with modern, otherwise so powerful medicine? Even here my local informants had an answer: because, ‘by definition’, all sorcery diseases (*kio'one*) were resistant to anything but traditional ways of treatment.

So, after years of intense contemporary kuru research with excellent scientific results, the Fore still seemed to stick to their ancient opinions about this sad and complicated subject. However, at the end of our candlelight session, it was, as a moderate compromise, taken into account that Masta Galten's ‘kuru germs’ might be hidden in the sorcerer's stone: ‘Em, liklik toktok i pinis nau’ (enough said).

In 1984, my illustrated book *Toktok* about my experiences in the highlands and Sepik regions of Papua New Guinea was published (Stöcklin 1984); among the photographs in the book are two of kuru patients. It was reprinted in the following year and in 2004 a revised and enlarged third edition came out (Stöcklin 2004).

## 3. 2007

Though I am very happy to learn that probably no new cases of kuru are to be expected in future, I have, strangely enough, some nostalgic feelings in regard to a most fascinating ‘kuru time’—with all the adventures, stories, intellectual acrobatics and long-lasting friendships resulting from many extraordinary situations in a beloved world at the edge of the Stone Age.
